# Factors affecting the concordance between orthologous gene trees and species tree in bacteria

**DOI:** 10.1186/1471-2148-8-300

**Published:** 2008-10-30

**Authors:** Santiago Castillo-Ramírez, Víctor González

**Affiliations:** 1 Programa de Genómica Evolutiva, Centro de Ciencias Genómicas, Universidad Nacional Autónoma de México, Apartado Postal 565-A, CP 62210, Cuernavaca, Morelos, México

## Abstract

**Background:**

As originally defined, orthologous genes implied a reflection of the history of the species. In recent years, many studies have examined the concordance between orthologous gene trees and species trees in bacteria. These studies have produced contradictory results that may have been influenced by orthologous gene misidentification and artefactual phylogenetic reconstructions. Here, using a method that allows the detection and exclusion of false positives during identification of orthologous genes, we address the question of whether putative orthologous genes within bacteria really reflect the history of the species.

**Results:**

We identified a set of 370 orthologous genes from the bacterial order *Rhizobiales*. Although manifesting strong vertical signal, almost every orthologous gene had a distinct phylogeny, and the most common topology among the orthologous gene trees did not correspond with the best estimate of the species tree. However, each orthologous gene tree shared an average of 70% of its bipartitions with the best estimate of the species tree. Stochastic error related to gene size affected the concordance between the best estimated of the species tree and the orthologous gene trees, although this effect was weak and distributed unevenly among the functional categories. The nodes showing the greatest discordance were those defined by the shortest internal branches in the best estimated of the species tree. Moreover, a clear bias was evident with respect to the function of the orthologous genes, and the degree of divergence among the orthologous genes appeared to be related to their functional classification.

**Conclusion:**

Orthologous genes do not reflect the history of the species when taken as individual markers, but they do when taken as a whole. Stochastic error affected the concordance of orthologous genes with the species tree, albeit weakly. We conclude that two important biological causes of discordance among orthologous genes are incomplete lineage sorting and functional restriction.

## Background

Fitch coined the term orthologous genes to describe genes whose phylogenies represent the phylogeny of the species [1,2]. Classically, gene orthology is established by comparing the phylogenetic tree obtained from the gene in question with that for the reference species. As bacterial comparative genomics deal with large amounts of data, requiring extensive computational power and time, sophisticated phylogenetic analysis cannot be easily automated. Thus, most of the studies in this area have used sequence similarity approaches to infer orthology. The reciprocal best hits (RBH) and single gene families (SGF) approaches are the two most common bioinformatic techniques used to infer orthology in bacterial comparative genomics. However, both horizontal gene transfer (HGT), a very pervasive force among bacteria [3-6], and duplications with subsequent differential loss of orthologous genes (DSDL), may result in the misidentification of orthologous genes (false positives) whenever RBH or SGF are used. Moreover, even using *bona fide *orthologous genes and phylogenetically robust methods such as maximum likelihood, incorrect phylogenetic reconstructions may occur when inadequate substitution models are employed [7]. When phylogenetic inference is performed with proteins, inconsistencies may arise due to the use of an incorrect amino acid substitution matrix, or not taking into account for rate variations across sites or variation in the observed amino acid frequencies [8]. Even genome-scale analyses may be susceptible to systematic error when model selection is omitted or a poor model is chosen, particularly when divergence among genes is high. Furthermore, in the case of single markers, individual genes may be affected by stochastic error related to gene size.

*Gammaproteobacteria *and *Alphaproteobacteria *have been used as model organisms for examining whether a prokaryotic phylogenetic tree can be confidently inferred using many orthologous genes [4,5,9,10]. Phylogenetic concordance among virtually all (203 out of 205) of the selected gene families was found in the case of *Gammaproteobacteria *[10]. However, another study of the same data set determined that 10% of these families had been horizontally transferred and that too little phylogenetic signal was evident in the rest of the families [5]. More recently, it was found that only three out of 200 orthologous genes manifested the topology of the species tree, while 29% of the data set rejected the species tree [11]. In the case of *Alphaproteobacteria*, around 77% of the gene trees inferred from SGF manifested no significant differences with the proposed supertree, which was inferred from all the gene trees, and 76 gene trees were identical to this supertree [4]. In another study, although concatenated alignments indicated a robust tree for the *Alphaproteobacteria*, no two phylogenies obtained from individual families were alike [12]. This apparent incongruence among the trees derived for individual genes may be at least partly due to artefactual phylogenetic reconstruction. Notably, most of these studies did not undertake model selection for individual genes, but instead used a single matrix for all analyses. This may represent a significant flaw, as a recent study in *Proteobacteria *found that, depending on the genes studied, the use of different amino acid matrices is required [8]. However, it is also possible that false positives have caused distortions in some of the prior studies (i.e. the families that rejected the species tree could be subject to HGT and/or DSDL).

Here, we use a strict strategy to infer orthology. First, we establish a RBH approach that applies a higher threshold than regular RBH approaches; an E-value of 10e-12 is used, along with the requirement that the hits align across least 50% of their length. We then use confidence sets of gene trees and an observed sister group relationship to rule out false positives. In this study, we address the question of whether single bacterial orthologous genes, as defined by our strategy, reflect the history of the species. The number of genes in common among species and phylogenetic signal decrease as phylogenetic distance increases; thus, we avoid signal erosion and reduction in the numbers of genes by focusing on a group whose members are separated by only moderate phylogenetic distances. A previous genomic timescale study of prokaryotes estimated that *Caulobacter crescentus *diverged from some species belonging to the order *Rhizobiales *about 1.5 billion years ago, whereas *Alphaproteobacteria *and *Gammaproteobacteria *were estimated to diverge about 2 billion years ago [13]. Here, we use members of the *Rhizobiales *order to make reliable phylogenetic inferences and by applying model selection for each phylogeny we try to avoid artefactual reconstructions.

Our results indicate that orthologous genes manifest a great diversity of phylogenies, and this diversity implies different topologies and models of evolution, as well as an ample level of divergence. The concordance of the orthologous gene trees with the best estimate of the species tree is affected by stochastic error related to gene size, although weakly and the effect is not distributed evenly among functional categories. While the individual phylogenies inferred from orthologous genes are not found to reflect the exact history of the species, the majority of the bipartitions composing the individual phylogenies do reflect such history. The nodes presenting greatest discordance are those defined by the shortest internal branches in the best estimate of the species tree. We see a clear bias concerning the functional categories of the orthologous genes, and this influences their degree of divergence. These results indicate that both functional restriction and incomplete lineage sorting are important factors driving discordance.

## Results

### The initial set of potential orthologous genes and a probable species tree

**Figure 1:**
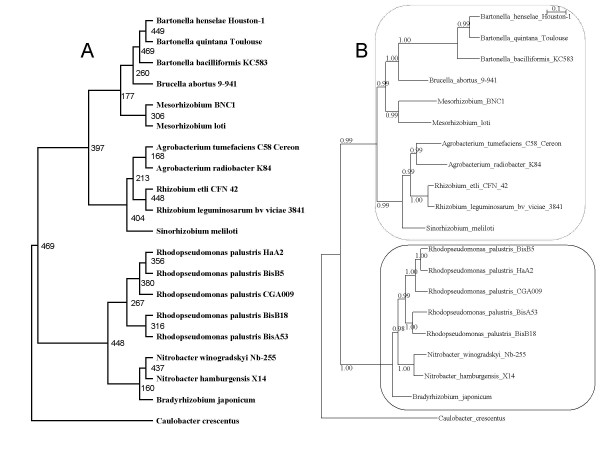
**The superalignment Bayesian phylogeny (SBP469) and the consensus tree (CT469) constructed from 469 potential orthologous genes**. A: CT469, the numbers to the right of the internal branches indicate the number of orthologous genes that contain the group defined by that internal branch. B: SBP469, the numbers on the branches give the posterior probability of the group. The sister group relationship between groups 1 and 2 is denoted by the dashed line (group 1) and the thick line (group 2). The scale bar denotes the estimated number of amino acid substitutions per site.

The RBH method was used to define an initial set of potential orthologous genes (see methods), yielding 469 candidates. A multiple sequence alignment and phylogeny were constructed for each orthologous gene (see methods). We then used these potential orthologous genes to deduce a probable species tree that helped us refine the set of potential orthologous genes. A consensus tree (469CT) was produced (Figure 1a) using the 469 phylogenies. By concatenating all the individual alignments, a superalignment was created and Bayesian and maximum parsimony phylogenies were inferred. Both methods yielded the same topology; for convenience, the superalignment Bayesian phylogeny (469SBP; Figure 1b) was used for subsequent analyses. The topologies of the 469SBP and 469CT were almost identical, differing only in the position of *Bradyrhizobium japonicum*. The genera *Nitrobacter *and *B. japonicum *were grouped together under 469CT, excluding the genus *Rhodopseudomonas*, whereas *Nitrobacter *and *Rhodopseudomonas *clustered together under 469SBP, excluding *B. japonicum*. Under 469CT, the group comprised of *Nitrobacter *and *B. japonicum *had the smallest presence among single gene phylogenies, being contained in only 160 out of the 469 individual phylogenies.

### Ruling out falsely positive orthologs

**Figure 2:**
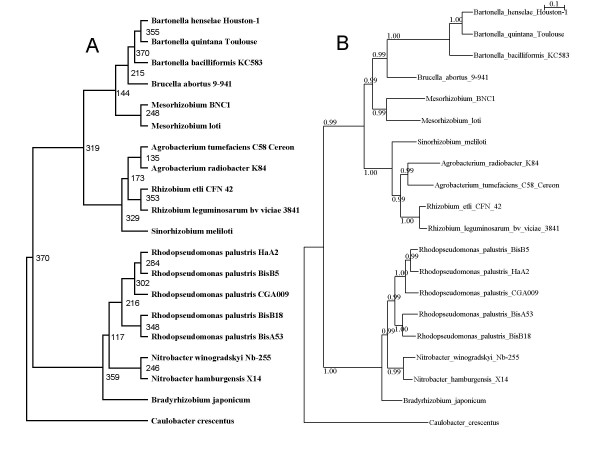
**The superalignment Bayesian phylogeny (SBP370) and the consensus tree (CT370), created from the 370 potential orthologous genes filtered from the larger data set**. A: CT370, the numbers to the right of the internal branches indicate the number of orthologous genes that contain the group defined by that internal branch. B: SBP370, the numbers on the branches give the posterior probability of the group. The scale bar denotes the estimated number of amino acid substitutions per site.

Even though our RBH approach was stringent, in that we applied BLAST searches with an E-value of 10e-12 and required proteins to align along at least 50% of their length, false positives may still result. In order reduce the risk of false positives, we inferred confidence sets for the 469 alignments. The 469SBP and 469CT topologies were tested for all alignments (see methods). The most accepted topology was 469SBP, which could not be rejected by 432 potential orthologous genes, whereas only 400 potential orthologous genes did not reject the 469CT topology. As the superalignment only accepted the 469SBP topology, we ruled out the 37 potential orthologous genes that rejected this topology. The 469SBP topology included two very well supported sister groups (Figure 1b, dashed and thick lines). The first group, which was supported by a posterior probability of 0.99, comprised *Sinorhizobium meliloti *and the genera *Rhizobium*, *Agrobacterium*, *Mesorhizobium*, *Bartonella*, and *Brucella abortus *9–941. The second group, which had a posterior probability of 1.0, comprised the genera *Rhodopseudomonas *and *Nitrobacter*, and *B. japonicum*. This sister group relationship was used to screen the 432 potential orthologous genes that accepted the 469SBP topology, ruling out all potential orthologous genes that contradicted the sister group relationship. This filtering yielded a set of 370 potential orthologous genes, which was expected to not include false positives. New versions of the superalignment Bayesian phylogeny (370SBP) and consensus tree (370CT) were constructed using these 370 orthologous genes (Figure 2). Both analyses yielded the same topology as that found for 469SBP. Both 469SBP and 370SBP had similar branch lengths (see Figure 1b and Figure 2b); however, because 370SBP arguably contained no false positives, it represented the most accurate approximation of the species history. As many authors have used lower thresholds when identifying orthologous genes, we lowered the E-values to 10e-9 and 10e-6 and applied the two filters to see how many orthologous groups could be rescued under these E-values. With E-values of 10e-9 and 10e-6, we rescued 31 and 38 more groups, respectively, compared to the earlier analysis. This indicates that the majority of groups had E-values equal to or greater than 10e-12 (i.e. only 38 more groups were found when the E-values was lowered from 10e-12 to 10e-6). Because the difference between the use of E-values of 10e-6 and 10e-9 was only eight more groups, we further examined the former (38 rescued groups) using the filters described above. Of the 38 groups, 20 were ruled out by one or both of the filters. Thus, for the 38 groups that were picked up by an E-value of 10e-6 but not 10e-12, almost 50% were ruled out by the utilized filters. Notably, however, when both filters were applied, the percentage of rejection was almost equal for the data sets obtained using E-values of 10e-12 and 10e-6, with 370 out of 469 groups (79%) and 390 out of 507 groups (77%), respectively, passing both filters.

### The identified orthologous genes had good phylogenetic content and substantial support

We used likelihood mapping analysis to analyze the phylogenetic content of the data set (see methods). Recognizing that a data set provides phylogenetic signal if it contains a high percentage of resolved quartets [14], we first determined the percentage of resolved quartets for each gene. The mean value of resolved quartets for all orthologous genes was 90.9% [standard error (SE), 1.32%; mode, 91.5%]. Even if all quartets are completely resolved, it is possible that the quartet-puzzling tree is not completely resolved when the quartets are not compatible with each other [14]. In our data set, only 82 orthologous groups presented a completely resolved puzzling tree; the groups yielding incompletely resolved puzzling trees comprised principally *B. japonicum *and the genus *Agrobacterium*. The superalignment had all quartets resolved and its puzzling tree was completely resolved. As a measure of support for our phylogenies, we calculated the median bootstrap value across the whole phylogeny, and then calculated the mean of the median values. The mean of the median values was 77 (SE = 4). These findings indicate that the identified orthologous genes had sufficient phylogenetic signal and substantial support.

### Almost every orthologous gene had a unique topology, and the most common topology was not that of 370SBP

In order to evaluate the diversity of evolutionary histories among the orthologous genes, we determined the number of different topologies. Approximately 93% of the orthologous genes presented unique topologies, for a total of 346 different topologies. Only two orthologous genes, namely ATP-dependent Lon protease (COG0466) and DNA-directed RNA polymerase beta subunit (COG0085), yielded the SBP370 topology. Unexpectedly, the most frequent topology (shared by six orthologous genes) was that of 469CT.

### Most bipartitions were in agreement with the 370SBP topology

In order to present a full account of phylogenic diversity, we examined the number of common bipartitions between the species tree and all the individual phylogenies. A bipartition represents the division of a phylogeny into two parts connected by a single internal branch; this divides the phylogeny into two groups but does not consider the relationships within each of the groups. The total number of different possible bipartitions for 20 taxa is 524,267; however, we only identified 254 different bipartitions in the individual phylogenies examined in the present study. The majority of bipartitions were in agreement with the 370SBP topology (71.5% of all observed bipartitions did not contradict this topology). Both 370CT and 370SBP yielded the same topology, thus they also shared the same bipartitions. Subsequently, 370CT reflected the frequencies of the bipartitions of 370SBP for the individual phylogenies. The frequencies of those bipartitions were not evenly distributed. There were only two cases where the nodes or bipartitions were supported by all of the orthologous gene trees. The separation of *Caulobacter crescentus *from the rest of the species represented one of these, while the other was the segregation of genus *Bartonella *from the other species (see Figure 2a). The two least frequently encountered bipartitions defined the genus *Agrobacterium *(supported by 135 phylogenies; see Figure 2a), and the group formed by *Rhodopseudomonas *and *Nitrobacter *but excluding *B. japonicum *(supported only 117 phylogenies). In addition, the branches that defined these two bipartitions/groups in 370SBP represented the second and third shortest branches across the whole phylogeny. Next, to estimate the similarities between each orthologous gene tree and the best estimate of the species tree, we calculated the percentage of common bipartitions between each orthologous gene tree and 370SBP (see methods). More than 90% of the 370 orthologous gene trees had more than 50% of their bipartitions in common with 370SBP. The mean percentage of common bipartitions among all orthologous genes was 71.76% and the mode was 76%. Thus on average, more than 70% of the bipartitions in each orthologous gene tree were also present in 370SBP.

### The larger the gene size, the higher the percentage of bipartitions in common; as a branch in the species tree grew larger, its bipartition frequency increased

To assess whether the stochastic error related to gene length affected the percentage of bipartitions in common, we tested for correlation between gene size and the percentage of common bipartitions. We found a weak but significant correlation (p < 0.00001; coefficients of correlation and determination, 0.39 and 0.15, respectively). This suggests that longer genes shared a higher percentage of bipartitions in common with the species tree. We also determined the correlation between the number of phylogenies that supported a bipartition in 370CT and the length of that branch in 370SBP. The coefficient of correlation was 0.624 and the coefficient of determination was 0.384 (p < 0.01), suggesting that longer branches defined groups (bipartitions) among a greater number of orthologous gene trees.

### Multiple best-fit protein models were selected

**Figure 3:**
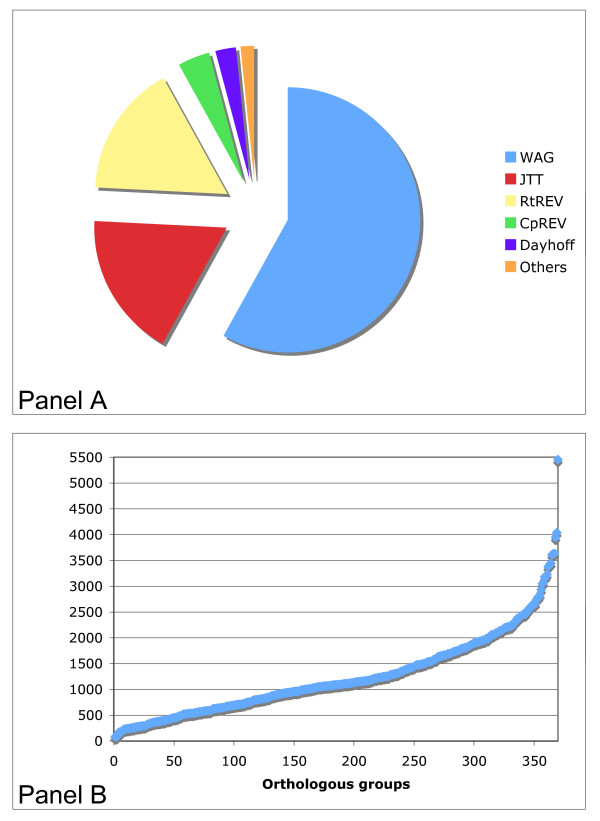
**The models of evolution selected by the orthologous genes**. The Akaike criterion of information was used to select the models of evolution. A: The different amino acid matrices selected. B: Difference between the best and worst models. The genes were ordered from lowest to highest with regard to the differences in the log likelihood values. Differences are positive because the worst models are more negative.

We then used the Akaike information criterion to allow each orthologous gene to select a model of protein evolution (see methods). The WAG matrix represented the most selected substitution model (selected by 58% of genes), followed by the JTT matrix (selected by around 18% of genes) (Figure 3a). Only four out of the eight selected models were chosen by more than 10 orthologous genes (Figure 3a). Although no single matrix was chosen for all genes, the preferred matrixes comprised a relatively small set. All of the orthologous genes had to be corrected for among-site rate variation. In addition, 68% also required correction concerning the frequencies of amino acids, and 40% were shown to have a proportion of invariable sites. To confirm that model selection improved our results, we examined the difference of the log likelihood values between the best and the worst models, according to the Akaike information criterion (where a high difference indicates an improvement). Approximately 56% and 85% of the genes showed differences higher than 1000 and 500, respectively, indicating that model selection improved our results (Figure 3b).

### Orthologous genes were functionally biased

We used the COG database [15] to functionally categorize (see methods) the identified orthologous genes into the four broad categories of this database. The frequency distributions of the functional categories differed significantly between our data set and that of the COG database (chi-square test p < 0.0005), indicating that the identified orthologous genes were functionally biased. The most common category in our data set, comprising 34% of the identified genes, was that of "Information Storage and Processing;" in contrast, most common category throughout the COG database was the "Poorly Characterized" category, which comprised 40% of the database (Table 1). On the flip side, the least frequent category in the COG database was that of "Information Storage and Processing" (15% of genes), while that in our data set was the "Poorly Characterized" category (12% of genes) (Table 1). In order to analyze the congruence among these broad categories, superalignment Bayesian phylogenies and consensus trees were constructed for each category. The superalignment Bayesian phylogenies for all four categories indicated the 370SBP topology. The consensus trees obtained for the "Information Storage and Processing" and "Cellular Processes and Signaling" categories also indicated the 370SBP topology, whereas the consensus trees for the "Metabolism" and "Poorly Characterized" categories differed from one another and from the 370SBP topology. The consensus tree obtained for the "Metabolism" category revealed a topology identical to that of 469CT (Fig 1a), while that for the "Poorly Characterized" category manifested the same discordance and, in addition, the genus *Agrobacterium *did not form a monophyletic group. These two points of discrepancy contradicted the two least common bipartitions in 370CT (the ones defined by the shortest internal branches in 370SBP). The correlation between gene size and the percentage of common bipartitions differed among the functional categories (Table 1); the "Poorly Characterized" category had the strongest correlation (coefficient of correlation, 0.49), while "Metabolism" had the weakest (non-significant) correlation (0.15, p = 0.072) (Table 1).

**Table 1:** Functional classification and percentage of genes in each functional category

Category	This data set	COG database	Stochastic error
Information storage and processing	34%	15%	0.36
Cellular processes and signaling	24%	18%	0.37
Metabolism	31%	28%	0.15*
Poorly characterized	12%	40%	0.49

Percentage of orthologous genes and COG database genes distributed across the four broad functional categories. The fourth column shows the correlation between gene size and the percentage of common bipartitions for each category. * This correlation was not significant.

### There was a wide variation in total phylogeny length

**Figure 4:**
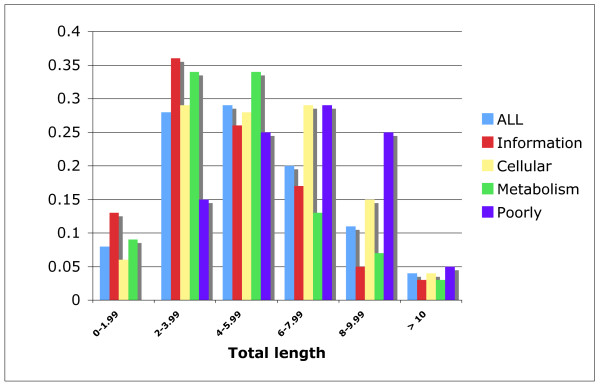
**Total phylogeny lengths**. As a measure of divergence we used the total phylogeny length, which is expressed as the estimated number of substitutions per site per phylogeny. This analysis was undertaken across the whole confidence set of orthologous genes (blue bars) as well as for the genes divided into four broad categories. Abbreviations are as follows: Information (red bars), the "Information Storage and Processing" category; Cellular (yellow bars), the "Cellular Processes and Signaling" category; Metabolism (green bars), the "Metabolism" category; Poorly (purple bars), the "Poorly Characterized" category.

To test for variation in the level of divergence within the set of orthologous genes, the total phylogeny length was determined for each individual phylogeny (see methods). We observed significant variation among the total lengths of the phylogenies (coefficient of variation, 53%), with a mean total length of 5.2 expected substitutions per site per phylogeny. Most of the phylogenies had between four and six expected substitutions per site per phylogeny (around 28%), followed by those having between two and four expected substitutions per site per phylogeny (almost 27%) (Figure 4, blue bars). When we tested whether the level of divergence was the same among the functional categories, we found significant differences among the categories (Kruskal-Wallis test, p < 0.0005). The "Poorly Characterized" category had the most diverged orthologous genes, with most genes (28%) having six to eight expected substitutions per site per phylogeny (Figure 4, purple bars). In contrast, the "Information Storage and Processing" and "Metabolism" categories had the least diverged orthologous genes, most of which fell into the range of between two and four expected substitutions per site per phylogeny (Figure 4, red and green bars, respectively). These observations suggest that the divergence of orthologous genes in this species appears to vary by functional class.

## Discussion

In this study, our goal was to test whether orthologous genes reflect the history of the species. To answer this question, we selected a monophyletic group having moderate phylogenetic distances (allowing us to make a reliable phylogenetic inference). We obtained an initial data set of possible orthologous genes using the reciprocal RBH technique, and further used two filters to infer gene orthology, thereby avoiding the inadvertent inclusion of DSDL and/or HGT. These filters excluded more than 20% of the initial data. The superalignment phylogenies and the consensus tree did not agree with one another when the initial data set was used. Once the false positives had been ruled out, however, both approaches produced the same tree. Thus, our results suggest that approaches using only computational definitions of orthology (e.g. RBH or SGF) can produce a considerable number of false positives, which contribute to disagreements among phylogenetic results.

As in other studies involving recently evolved groups [4,9,10], we found that orthologous genes had very good phylogenetic content. This set presented a strong vertical signal, indicated by the fact that both approaches used to infer the possible species trees revealed the same topology. Moreover, around 71% of the total bipartitions agreed with the inferred species trees. Therefore as a whole, our selected orthologous genes had a very strong vertical signal and manifested a tree-like organismal history. Other studies in *Alphaproteobacteria *and *Gammaproteobacteria *[4,9-12] reached the same conclusion when gene families were considered as a whole (e.g. superalignment and/or supertrees). In a recent study, a robust phylogeny for the *Alphaproteobacteria *was inferred [12], and the relationships revealed for the *Rhizobiales *group were equivalent to the species tree inferred in this study. We found a great diversity of topologies, many of which were well supported. Almost every orthologous gene revealed a distinct topology, yielding 346 different topologies. This is consistent with the findings of the previous study in *Alphaproteobacteria *[12], wherein none of the topologies from the individual genes were found to be equivalent. Furthermore, we found diversity not only in topology, but also in the models of protein evolution chosen by each of the orthologous genes. Eight amino acid substitution matrices were chosen, but only 4 had a frequency exceeding 10 genes. As in the previous study that identified a robust species tree for *Alphaproteobacteria *[12], the most frequent matrix identified among the orthologous genes was the WAG amino acid substitution matrix. The site rate variation and correction for amino acid frequency inequality parameters were strong performers in our study; all of the phylogenies described herein were based on models that accounted for site rate variation, and up to 68% of the phylogenies were corrected for inequalities in amino acid frequency.

The most frequently found topology differed from the species tree, although the only difference was the position of *B. japonicum*, which corresponded to one of the shortest branches in the best estimate of the species tree. Furthermore, only two orthologous genes yielded the species tree topology. These findings are in accordance with similar findings from other reports [11,12]. For instance, within *Gammaproteobacteria *only three out of 200 genes had the same topology as the reference tree [11]. These findings collectively suggest that most orthologous genes do not reflect the exact species tree when used as individual markers, and the most common topology can differ from the species tree. This is a significant point, because it suggests that even at moderate phylogenetic distances (where phylogenetic inference is reliable when adequately performed), neither a single orthologous gene nor the most common topology can be used to reconstruct the exact history of the species. However, even though only two of the orthologous gene trees manifested the species tree topology, all the orthologous gene trees together shared an average of 71% of their bipartitions with the species tree. Thus, the majority of bipartitions composing the orthologous gene trees in this study reflected a large part of the species history. Nevertheless, individual orthologous gene trees were not evenly distributed with regard to the species tree bipartitions. Only the genus *Bartonella *and the separation of the ingroup from outgroup occurred in all of the individual phylogenies. The two bipartitions that showed the smallest representation among the individual phylogenies were two of the three shortest internal branches in the species tree (see Figure 2b), and involved the placements of *B. japonicum *and the genus *Agrobacterium*.

Gene length also emerged as a factor influencing discordance in our study, both at the level of the species tree and for the single orthologous gene trees. Even though the correlation was weak, the phylogenies of longer genes had more bipartitions in common with the species tree. This agrees with a recent study analyzing *Alphaproteobacteria*, which concluded that part of the problem with inferring phylogenies from individual genes resulted from insufficient information content, due to the short length of the genes [12]. Notably, however, this correlation was not equal across all functional categories; the "Poorly Characterized" category presented the strongest correlation, while the "Metabolism" category did not show significant correlation. This suggests that, where possible, it is better to choose longer orthologous genes from the "Poorly Characterized" category. On the other hand, a stronger correlation was found between bipartition frequency among the individual phylogenies and the internal branches of the species tree (where the least common bipartitions were defined by the shortest internal branches). Therefore, as more changes accumulate in a branch that defines a group in the species tree, more of the individual orthologous gene trees will reflect this group. This implies that even for species trees, special attention should be paid to the shortest internal branches, which will tend to be more problematic.

There are several non-biological causes that could cause discordance, such as imperfect sequence alignment, stochastic error related to gene length (discussed above), and model violations. We feel that model violations are not the main source of incongruence in the present study, because each orthologous gene was allowed to indicate its own model of evolution and the phylogenies were constructed using models that accounted for site rate variation and (where necessary) corrected for amino acid frequency inequalities.

Incomplete lineage sorting has been recognized as a biological factor that can lead to discordance when phylogenies are inferred from genes [16,17], particularly where the internal branches of the species tree are short enough so that coalescence of gene lineages may occur more deeply than the speciation event. Degnan and Rosenberg showed that very short branches deep in a species tree comprising five or more species can lead to anomalous genes trees (AGT), i.e. gene trees that do not match the species tree [16]. Furthermore, the most probable gene tree can have a different topology from that of the species tree if multiple branch lengths are small enough in coalescent units [16]. Two trends lead us to believe that incomplete lineage sorting is one of the main causes of discordance among the orthologous genes examined in the present study. First, the two least common bipartitions from the individual orthologous gene trees involve two out of three of the shortest internal branches in the species tree (Figure 2). Second, the most common topology was not that of the species tree, but it only differed from the species tree in terms of the position of *B. japonicum*, which involves precisely one of the very short, deep, internal branches discussed above. Indeed, when we used the COAL [18] software to determine the probability of the genes trees that had the most common topology and the genes trees that had the species tree topology, given our best estimate of the species tree, although all the genes with the most common topology got the same very low probability, which was 0.00000000001, the probability got by the genes with the species tree topology was 0.00000000000. Thus, the most common topology appears to be an example of AGT.

It is common for orthologous genes to broadly indicate the history of the species, without reflecting it exactly. In the present case, this is not related to signal erosion because most of the orthologous genes studied herein had good phylogenetic content. Instead, we think that the type of function fulfilled by each gene influenced its ability to recover the true tree. We found that the orthologous genes recovered by our analysis were functionally biased, with genes of the "Information Storage and Processing" category representing 34% of the orthologous genes (as compared to 15% of the COG database), while the "Poorly Characterized" category represented only 12% of the orthologous genes (compared to 40% in the COG database). Furthermore, the level of divergence paralleled the functional bias, as the categories containing more orthologous genes were less diverged. The most diverged category was that of the "Poorly Characterized" genes, which contained a very few highly diverged orthologous genes and yielded a consensus tree that differed considerably from the species tree. To a certain extent, this aspect of functional restriction also relates to the discordance caused by incomplete lineage sorting. The "Metabolism" and "Poorly Characterized" categories were the most affected by incomplete lineage sorting, as their consensus trees differed precisely in those branches where incomplete lineage sorting was a factor. The "Poorly Characterized" category contained the most diverged orthologous genes, and was the most adversely affected by lineage sorting. It is reasonable to deduce that weak functional restrictions may have allowed this. Following the same logic, a category with highly conserved (i.e. functionally restricted) genes should be less affected by incomplete lineage sorting, as seen for the "Information Storage and Processing" category.

In conclusion, we observed that orthologous genes exhibited a great diversity of phylogenies, having different best-fit models of evolution, topologies, and degrees of divergence. Thus, almost no single orthologous gene by itself can reflect the exact history of the species. Notably, the most frequent topology did not match the species tree. Orthologous genes were affected by stochastic error relating to gene size, although this effect was relatively weak and was not evenly distributed across the functional categories. The most problematic clades were those defined by short internal branches, as these suffered from the effects of incomplete lineage sorting. The extent of these effects depended on the functional restrictions of the orthologous genes; for example, the "Information Storage and Processing" category appeared to be refractory to this process, whereas the "Poorly Characterized" category was more highly affected. When we used as many markers as possible, however, we could achieve a good reconstruction of the species history. For instance, when we employed superalignment, even the "Poorly Characterized" category indicated the topology of the species tree. Thus, when taken as a complete set, orthologous genes have a great capacity for depicting the history of a species.

## Methods

### Genomes used

We used the complete proteomes of 25 *Alphaproteobacteria *(see additional file 1), including 24 belonging to the *Rhizobiales *order and the genome of *Caulobacter crescentus*, which was used as outgroup. All of the genomes, except those of *Agrobacterium radiobacter *K84 and *Agrobacterium vitis *S4, were downloaded in February of 2007 from the NCBI ftp site. Those of *Agrobacterium radiobacter *K84 and *Agrobacterium vitis *S4 were downloaded from Agrobacterium.org http://depts.washington.edu/agro/.

### Defining orthologous groups

#### Using the RBH approach to identify possible orthologous groups

As *B. quintana *strain Toulouse has the smallest proteome out of all the species considered herein, this strain was used as the reference genome to establish an RBH approach. Each of the 1142 proteins of *B. quintana *strain Toulouse were compared with the proteomes of the other strains, using BLAST [19] with an E-value cutoff of < 1.0e-12. We retained all cases where a protein of *B. quintana *strain Toulouse had a bidirectional best hit in each of the other proteomes, and the proteins aligned along at least 50% of their lengths.

The above analysis yielded 469 groups (potential orthologs). Each of these possible orthologous groups were aligned using MUSCLE [20] with the default parameters. The best model of amino acid substitution for each alignment was determined using ProtTest [21], and the most likely phylogeny was constructed using PHYML [22] with 100 non-parametric bootstrap replicates. The gamma shape parameter and the proportions of invariable sites were estimated by maximizing the likelihood of the phylogeny. Likelihood mapping analysis was carried out to determine the phylogenetic content for every individual alignment, using PUZZLE [14,23].

#### Excluding redundant species

In our preliminary analyses, we noted that the genera *Agrobacterium *and *Brucella *contained species that showed minimal divergence. As a result, many possible orthologous groups manifested identical protein sequences for certain species belonging to *Agrobacterium *and/or *Brucella*. In order to exclude redundant species, we used PUZZLE to establish maximum likelihood matrices for the 469 alignments, taking into account among-site rate variation [14,23]. We then took the mean of the maximum likelihood distance between any two species; if two species had a mean distance equal to or less than 0.05, one of these was excluded. Five species were removed (marked with asterisks in additional file 1). We then established new alignments, model selection, and phylogenies without the excluded species.

#### Ruling out false positives

Two filters were used to eliminate false positives. The first filter consisted of using confidence sets to assess whether the differences in topology between the probable species trees (see below) and individual gene trees exceeded those expected to occur by chance. We used expected likelihood weighting [23], which provides a simple and intuitive method for making multiple comparisons of models and constructing corresponding confidence sets. This test has the benefit of being less conservative than the SH test [23]. The topologies tested included the superalignment Bayesian topology and the consensus tree topology (see below). PUZZLE was used to carry out this test for each of the 469 alignments, as well as for the superalignment (see below). The 469SBP typology (see Figure 1b) contained a sister group relationship between the group comprising *Sinorhizobium meliloti*, *Brucella abortus *9–941 and the genera *Rhizobium*, *Agrobacterium*, *Mesorhizobium*, and *Bartonella *and that comprising the genera *Rhodopseudomonas*, *Nitrobacter*, and *Bradyrhizobium japonicum*. The presence of this sister group relationship was used as the second filter; we used PAUP* 4.01 b10 [24] to see whether each of the 432 potential orthologous genes that passed the first filter had phylogenies manifesting the two sister groups. We then used likelihood mapping analysis (applied through PUZZLE) to determine the phylogenetic content for each of the remaining orthologous genes; the number of resolved quartets was counted for each gene, and then a mean and SE were calculated for the entire set.

### Two approaches for establishing a probable species tree

#### Superalignment approach

A superalignment was created by concatenating the 469 individual alignments. Two phylogenies were derived. The first was undertaken with maximum parsimony, using PAUP* 4.01 b10 [24] with random addition of sequences and tree bisection reconnection. The second phylogeny was created using MrBayes v3.1.2 [25], allowing the MCMC sampler to explore all of the fixed-rated amino acid models included in MrBayes. The number of rate categories for gamma distributions was set to four, with an allowance for a proportion of sites to be invariable. Due to the computational burden, we performed a single run with four chains, for 500,000 generations. Trees were sampled every 500 generations, 25% of all generations were removed as burn-in, and a consensus was taken. Once the candidate orthologous genes had been filtered for removal of false positives, we generated a second Bayesian phylogeny from the remaining 370 genes, using the same specifications as above. Because we ran only one run, for each Bayesian phylogeny, we could not use the standard deviation of the split frequencies, instead we examined the log likelihood values. For both superalignments, these values stabilized very soon and started to fluctuate within a very narrow range. In additional file 2 we plotted the log likelihood values of the second phylogeny.

#### Consensus tree approach

A consensus tree was created from all 469 phylogenies using CONSENSE [26]. Once the candidate orthologous genes had been filtered for removal of false positives, we generated a second consensus tree from the remaining 370 genes.

#### Topologies and bipartitions

The number of different topologies for the confidence set of orthologous groups was deduced using the Robinson and Fould distance (RFd), as calculated through application of TREEDIST [26]. The RFd indicates the number of bipartitions that are unique to one of two phylogenies being compared; the RFd equals zero when the two phylogenies have the same topology. The number and proportion of total bipartitions were determined using an *ad hoc *perl script that is based on inputting the consensus file generated from CONSENSE [26].

#### Percentage of bipartitions in common between the 370SBP and each individual phylogeny

We calculated the RFd between each individual phylogeny and the species tree and used it to determine the percentage of shared bipartitions. Each phylogeny had 17 bipartitions, and two phylogenies were considered in each comparison, for a total of 34 bipartitions in each comparison. The RFd reflected the number of bipartitions that were unmatched within the data set. For example, an RFd of four indicated that 30 bipartitions were shared. In order to establish the percentage of common bipartitions for each phylogeny, the number of shared bipartitions was divided by two, because two phylogenies were being considered. In our example this would be 30/2, which equals 15. Thus, 15 out of 17 (88%) of the bipartitions would be common to the two phylogenies. Therefore, the formula for establishing the percentage of common bipartitions is as follows:

Percentage of common bipartitions = ((34-RFd)/2) × 100

#### Functional assignment

We used the COG database [15] to undertake functional annotation across the four broad categories of "Information Storage and Processing," "Cellular Processes and Signaling," "Metabolism," and "Poorly Characterized." A few orthologous genes that had not been functionally assigned within the COG database were placed in the "Poorly Characterized" category. We excluded all orthologous genes that belonged to two or more broad categories. We chose this method because broad classification is less prone to error.

## Authors' contributions

SC-R conceived, designed, and performed the experiments. SC-R analyzed the data and wrote the manuscript. VG contributed materials and edited the manuscript. All authors read and approved the final manuscript.

## Supplementary Material

Additional file 1 Genomes used. Click here for file

Additional file 2 The log likelihood values of the 370SBP. Click here for file
