# Supplementary material for: Factors affecting the concordance between orthologous gene trees and species tree in bacteria
Source: BMC Evol Biol. 2008 Oct 30;8:300. doi: 10.1186/1471-2148-8-300 (PMC2614993; doi:10.1186/1471-2148-8-300)
Supplement: Additional file 1 — Genomes used. [file 1471-2148-8-300-S1.doc]

### Additional file 1. Genomes used

*Agrobacterium radiobacter* K84

**Agrobacterium vitis* S4

*Sinorhizobium meliloti*

*Rhodopseudomonas palustris* HaA2

*Rhodopseudomonas palustris* CGA009

*Rhodopseudomonas palustris* BisB5

*Rhodopseudomonas palustris* BisB18

*Rhodopseudomonas palustris* BisA53

*Rhizobium leguminosarum* bv viciae 3841

*Rhizobium etli* CFN42

*Nitrobacter winogradskyi* Nb-255

*Nitrobacter hamburgensis* X14

*Mesorhizobium loti*

*Mesorhizobium* BNC1

*Caulobacter crescentus*

**Brucella suis*1330

**Brucella melitensis* biovar Abortus

**Brucella melitensis*

*Brucella abortus* 9-941

*Bradyrhizobium japonicum*

*Bartonella quintana* strain Toulouse

*Bartonella henselae* Houston-1

*Bartonella bacilliformis* KC583

**Agrobacterium tumefaciens* C58 UWash

*Agrobacterium tumefaciens* C58 Cereon

* These species were excluded because they were determined to be redundant with another species in the set.
