# Supplementary figures and images for: Factors affecting the concordance between orthologous gene trees and species tree in bacteria
Source: BMC Evol Biol. 2008 Oct 30;8:300. doi: 10.1186/1471-2148-8-300 (PMC2614993; doi:10.1186/1471-2148-8-300)

## Slide 1
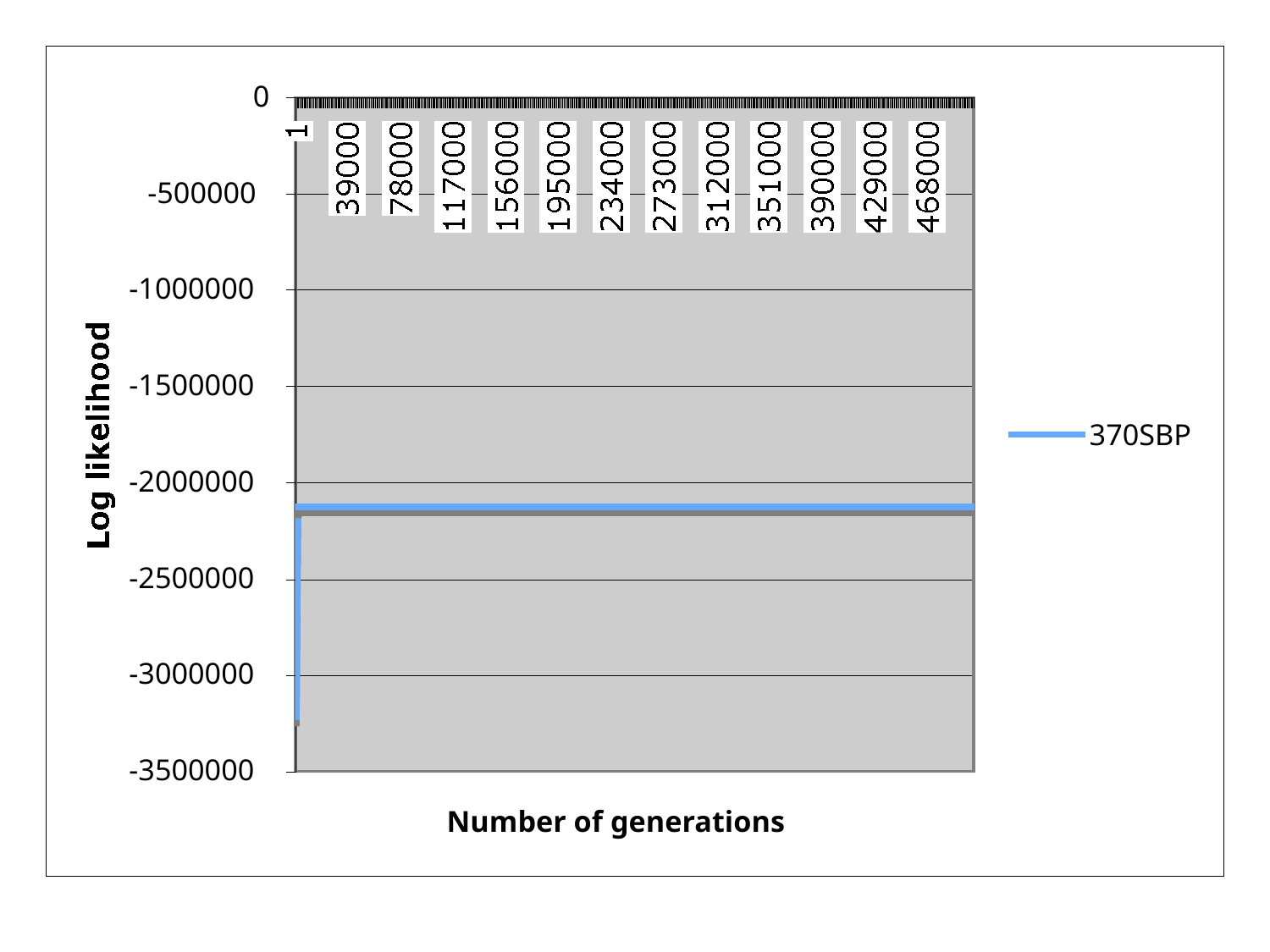

Supplement: Additional file 2 — The log likelihood values of the 370SBP. [file 1471-2148-8-300-S2.ppt]
